# Virtual care and COVID-19: A survey study of adoption, satisfaction and continuing education preferences of healthcare providers in Newfoundland and Labrador, Canada

**DOI:** 10.3389/fdgth.2022.970112

**Published:** 2023-01-25

**Authors:** Vernon R. Curran, Ann Hollett, Emily Peddle

**Affiliations:** Office of Professional & Educational Development, Faculty of Medicine, Memorial University of Newfoundland, St. John’s, NL, Canada

**Keywords:** virtual care, survey, healthcare providers, satisfaction, confidence, digital professionalism, continuing professional development

## Abstract

**Introduction:**

Virtual care has expanded during COVID-19 and enabled continued access to healthcare services. For many healthcare providers, the adoption of virtual care has been a new experience in the provision of healthcare services. The purpose of this survey study was to explore healthcare providers' experiences with virtual care during COVID-19.

**Methods:**

A web-based survey-questionnaire was developed by applying Rogers' theory of diffusion of innovation and distributed to healthcare providers (physicians, nurses and allied health professionals) in Newfoundland and Labrador, Canada to explore virtual care experiences, satisfaction and continuing professional development (CPD) needs. Analyses included descriptive statistics and thematic analysis of survey responses.

**Results:**

Fifty-one percent of respondents (*n* = 432) indicated they were currently offering virtual care and a majority (68.9%) reported it has improved their work experience. Telephone appointments were preferred over videoconferencing by respondents, with key challenges including the inability to conduct a physical exam, patients' cell phone services being unreliable and patients knowing how to use videoconferencing. Majority of respondents (57.5%) reported quality of care by telephone was lower than in-person, whereas quality of care by videoconferencing was equivalent to in-person. Main benefits of virtual care included increased patient access, ability to work from home, and reduction in no-show appointments. Key supports for adopting virtual care included in-house organizational supports (e.g., technical support staff), local colleague support, and technology training. Important topics for virtual care CPD included complying with regulatory standards/rules, understanding privacy or ethical boundaries, and developing competency and digital professionalism while engaging in virtual care.

**Discussion:**

Beyond the COVID-19 pandemic, virtual care will have a continuing role in enhancing continuity of care through access that is more convenient. Survey findings reveal a number of opportunities for supporting healthcare providers in use of virtual care, including CPD, guidelines and resources to support adaptation to virtual care provision (e.g., virtual examinations/assessments), as well as patient educational support.

## Introduction

Over the past several years, the world has been significantly affected by the spread of a novel pneumonia pandemic caused by severe acute respiratory syndrome coronavirus 2 (SARS-CoV-2), resulting in coronavirus disease (COVID-19). COVID-19 has had a considerable impact on healthcare systems worldwide, including the need to continue providing diagnosis, treatment, monitoring and follow-ups during the pandemic despite major infectious outbreaks and public health restrictions. Primary healthcare service delivery was initially challenged as healthcare services were disrupted due to inadequate personal protective equipment (PPE), lockdown^1^, and risk of infection spread to patients, healthcare providers and staff. Rapid deployment and adoption of virtual care has allowed healthcare providers to continue offering timely care while minimizing the risk of exposure for themselves and patients. As a result, most provincial healthcare systems in Canada responded to the emerging challenges associated with COVID-19 through rapid adoption of digital tools and technologies.

It has been suggested that virtual care technologies can be integrated into the healthcare system to maximize the efficiency of healthcare delivery (1, 2). Virtual care refers to the delivery of healthcare services digitally or at a distance using Information and Communications Technology (ICT) (3). In Canada, virtual care was used for control and triage during COVID-19, self and distance monitoring, treatment, and implementation of online health services. The use of virtual care during COVID-19 was believed to offer more timely care while minimizing exposure to protect healthcare providers and patients (3–7). By minimizing in-person visits and reducing face-to-face contact among physicians and patients, the use of virtual care may have helped to reduce virus transmission and protect healthcare providers from infection.

For many healthcare providers, the adoption and integration of virtual care into healthcare service provision during COVID-19 has been a new experience. During COVID-19 a variety of virtual care modalities have been used, with the most common types including synchronous and asynchronous appointments between patients and providers. Synchronous virtual care is communication which occurs live, including telephone and videoconferencing. Asynchronous is communication which does not occur live and may include email, patient portal messages and e-consults. Emerging research from the COVID-19 pandemic period suggests that virtual care has been beneficial for healthcare providers, patients and the general community (8). Adopting virtual care during COVID-19 has saved on costs associated with PPE and disinfecting healthcare spaces (4, 6, 9), and enabled patients to stay home who may have otherwise travelled to a hospital and incurred risk of unnecessary exposure (5–7). Virtual care adoption also enabled COVID-19 patients remaining at home to continue to receive follow-up and monitoring from providers (9).

Canada Health Infoway (an independent not-for-profit organization funded by the federal government) and the Canadian Medical Association (CMA) conducted the 2021 National Survey of Canadian Physicians to better understand the use of digital health and information technology among physicians (family physicians and specialists) practicing throughout Canada. Ninety-four percent (94%) of physicians surveyed reported offering virtual care in their practice. However, in-person visits were still very prevalent with half of patients (5 in 10) continuing to be seen in person by their physician (10). The types of virtual care offered included telephone care, video visits, and email/messaging. Very few respondents provided remote patient/home health monitoring using mobile or remote devices (10). In August 2021, the Canadian Institute for Health Information (CIHI) also released data showing the increase in virtual care use across several provinces during the COVID-19 pandemic (11). In February 2020, the percentage of physicians who had provided at least one virtual care service was 48%, and by September 2020 this had increased to 83%. For patients, the proportion of people receiving at least one virtual care service increased from 6% to 56% during this time period as well (11). Similarly, various hospitals in the United States during March and April of 2020 experienced a decrease of more than 80% of in-person visits in favor of virtual care (6).

Virtual care is believed to improve patient access, enabling quality and efficient care for patients (10). Appleton, et al. (12) found virtual mental healthcare to be as effective as in-person, while some authors have reported that shared decision-making processes with patient-centred care to be effective through virtual care during COVID-19, particularly so when using synchronous appointments (13–15). Generally, patients living in remote areas or with minor ailments respond positively to virtual care with reduction in travel and increased access by enabling the convenience of communicating with healthcare providers from their own home (7). Imlach, et al. (16) reported that 91% of patients in their survey study were satisfied with their experience with virtual care (e.g., telephone and video consultations), compared to 92% who expressed satisfaction with their in-person care visits.

Further understanding of the experiences, perceptions and potential continuing professional development (CPD) needs of healthcare providers is important in ensuring that appropriate support systems are in place to enable providers to adopt and use virtual care effectively and efficiently in their practices. Research suggests that a lack of training specific to virtual care tools and software is a challenge for providers. Lack of understanding and training can contribute to provider unwillingness to use virtual care, subsequently challenging virtual care adoption (4, 12, 17, 18). Adapting clinical approaches to patient care can also be challenging, more specifically the challenge of virtually examining patients (8, 10, 17). Providers could fear the implications of a misdiagnosis because of physical examination limitations and such a challenge could be a barrier for providers' in adjusting to the adoption and use of virtual care (4, 18). Integration of virtual care can also have an associated increase in administrative tasks for providers (7, 8, 18). It has been reported that virtual care adds additional workload on healthcare providers and its implementation tends to involve excess paperwork (18). The Canada Health Infoway and Canadian Medical Association (CMA) 2021 survey also highlighted the accessibility challenges that some patients and communities may face with respect to virtual care (10, 17). In particular, good internet access can be challenging for rural areas and can affect the quality of communication between providers and patients, subsequently effecting the patient experience (4, 8). Despite the potential advantages afforded by virtual care, it is important to understand how such technologies can also create a ‘digital divide’ that may exclude access for some patients. Multiple modes of health services delivery can promote greater health equality by not excluding certain patient groups.

Given the rapid introduction and adoption of virtual care modalities during COVID-19, as well as continued interest in how virtual care may complement healthcare provision into the future, further understanding of how virtual care was experienced by providers is imperative for ensuring appropriate supports are in place moving forward. We sought to explore the experiences, perceptions and satisfaction of healthcare providers in Newfoundland and Labrador, Canada with adoption and use of virtual care during COVID-19 through a survey questionnaire study. As explained below, our study was informed by Rogers' Innovation Diffusion Theory (19).

According to Rogers' theory of innovation diffusion, innovation is an idea, process, or a technology that is perceived as new or unfamiliar to individuals. Rogers' theory is one of the most popular theories for studying adoption of information technologies (IT) and has been applied in several studies to conceptualize the adoption of telehealth and virtual care (19–22). There are four main determinants of success of an IT innovation: communication channels, the attributes of the innovation, the characteristics of the adopters, and the social system (19). Specifically, the ‘attributes of an innovation’ include five user-perceived qualities: relative advantage, compatibility, complexity, trialability and observability. Relative advantage is the degree to which an innovation is perceived as better or improves upon existing practices, while compatibility describes the extent to which an innovation is consistent with the values, past experiences and needs of the potential adopter. The more an innovation integrates or coexists with these, the greater its prospects for adoption. Complexity involves the degree to which an innovation is perceived to be difficult to understand and use. Innovations with less complexity are more likely to be adopted more quickly. Trialability is the extent to which an innovation can be experimented with on a limited basis, whereas observability describes the degree to which the benefits of an innovation are visible to potential adopters. Roger's theory was applied in this study as the theoretical framework to explore the various factors influencing the adoption and choice of virtual care, benefits and barriers, and perceived enablers to adoption.

## Methods

A survey-questionnaire, provided in the **Supplementary Material,** was constructed by adapting aspects of several surveys from the peer-reviewed literature (23–25). As discussed, Roger's Innovation Diffusion Theory was applied as a theoretical framework and more specifically we employed the concepts underlying the attributes of the innovation - ‘complexity’, ‘relative advantage’ and ‘compatibility’ - in designing the survey questionnaire items (19). Some questions were also adapted from learning objectives of a Delphi Survey developed by the Association of Faculties of Medicine of Canada (26). The final survey included a combination of 34 closed and open-ended items to collect information on: background characteristics and demographics; current use of virtual care; satisfaction and confidence using virtual care; barriers and challenges of using virtual care; and CPD topics/needs.

Validation of the final survey items was undertaken using a two-step process. First, a draft of the survey was reviewed by an advisory committee comprising fifteen (*n* = 15) representatives of the various healthcare provider respondent populations and key local policy and program stakeholders in virtual care delivery. The questionnaire was reviewed item by item with committee members during a scheduled meeting, and items were removed or revised based on feedback surrounding the clarity and relevance of the items. Second, a final draft of the survey was reviewed and piloted with a sample of six (*n* = 6) healthcare providers representing the different provider groups, and a final web-based version of the survey was developed using the Qualtrics survey system.

E-mail messages with a link to the web-based survey were distributed to healthcare providers between January and March 2022, including a follow-up reminder 2–3 weeks later. E-mail invitations were distributed through provincial professional organizations for physicians, nurses, psychologists, social workers, occupational therapists, physiotherapists and speech language pathologists. Survey results were analyzed using the Statistical Package for the Social Sciences (SPSS 28.0). Methods of analysis included descriptive statistics (frequencies) and cross tabulations using Pearson's *x^2^* test to identify any significant differences between professional groups. Professional respondent categories were created to include: physicians, nurses, and allied health professionals. A thematic analysis was conducted on any open-ended question responses. The Newfoundland and Labrador Health Research Ethics Board (HREB) provided approval for this study, Reference # 2021.239.

## Results

The online survey was distributed to *N* = 7,679 health professionals (*n* = 1,382 physicians, *n* = 3,600 nurses, *n* = 300 psychologists, *n* = 1,700 social workers, *n* = 240 occupational therapists, *n* = 304 physiotherapists, and *n* = 153 speech language pathologists). The survey was completed by *N* = 1,013 respondents (response rate of 13.2%) (Supplementary Table S1). Four hundred and thirty-two (*n* = 432, 51.1%) respondents indicated they used virtual care during the COVID-19 pandemic. Respondents not offering virtual care indicated the main reasons were related to their ‘practice not promoting virtual care’ (*n* = 106, 25.7%) and ‘not trained on how to provide virtual care’ (*n* = 67, 16.2%). A higher percentage of allied health professionals reported ‘not trained on how to provide virtual care’, ‘scheduling issues’, and ‘lack of technical support’, whereas a higher percentage of physicians reported ‘I tried it and found it frustrating to use’ and ‘I don’t believe virtual care is an appropriate way to evaluate patients/clients’. Non-users also offered other reasons as well, with *n* = 42 respondents indicating ‘working in acute care, ER, hospital setting’ as a main reason for non-adoption.

**Figure 1:**
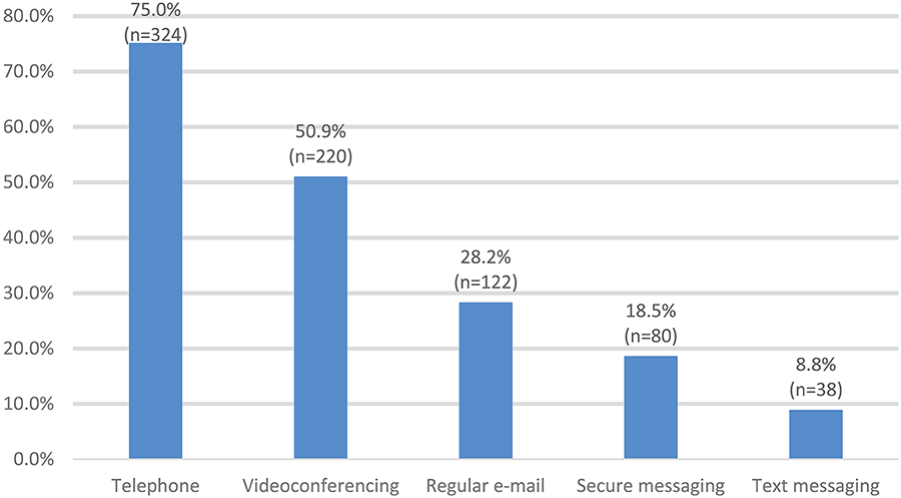
Types of virtual care currently offered.

Figure 1 summarizes the types of virtual care currently offered with the highest reported modalities being telephone (*n* = 324, 75.0%), videoconferencing (*n* = 220, 50.9%), and e-mail (*n* = 122, 28.2%). A higher percentage of physicians reported offering telephone appointments, while allied health professionals reported offering videoconferencing appointments, secure messaging, and regular e-mail (*p* < .05). Two hundred and three respondents (*n* = 203, 56.2%) indicated they preferred using telephone appointments vs. 43.8% (*n* = 158) preferring videoconferencing. A higher percentage of physicians preferred telephone appointments compared to allied health professionals preferring videoconferencing (*p* < .05). The following results are summarized according to Roger's key attributes of ‘complexity’, ‘relative advantage’ and ‘compatibility’.

### Complexity

Roger's attribute of ‘complexity’ is described as the degree to which an innovation is perceived to be difficult to understand and use, with greater complexity being a larger barrier or challenge to adoption (19). Several questions on the survey were designed to examine respondents' experiences or perceptions of reasons for not adopting and using virtual care or different virtual care modalities (e.g., survey items # 10, 14, 15, 16). The key reasons rated by a higher proportion of respondents across professional groups for not conducting videoconferencing appointments were ‘patients/clients do not own necessary equipment (e.g., computer, webcam)’ (*n* = 86, 19.9%), 95% CI [16.2, 23.8], ‘patients/clients do not know how to use videoconferencing’ (*n* = 76, 17.6%), 95% CI [14.1, 21.4], and ‘prefer to conduct telephone appointments’ (*n* = 71, 16.4%) 95% CI [13.2, 20.0] (Table 1). When the ratings for each reason were compared by professional group using Pearson's *x*^2^ test, the results indicated a significant difference at the *p* < .05 level for several of the reasons. A higher percentage of physicians (family physicians and specialists) reported preferring to conduct telephone appointments and in-person appointments, as well as indicating that logistics surrounding videoconferencing were too complicated, they did not own the necessary equipment, they did not have adequate internet connection, and patients/clients did not know how to use videoconferencing nor own necessary equipment.

**Table 1:** Reasons for not conducting videoconferencing appointments by professional group.

Reasons for not conducting videoconferencing appointments**	All Respondents (*n* = 432)***	Physicians (*n* = 107)	Nurses (*n* = 193)	Allied Health (*n* = 126)	
	*n* (%)	*n* (%)	*n* (%)	*n* (%)	Sig.
Patients/clients do not own necessary equipment (e.g. computer, webcam)	86 (19.9%)	34 (31.8%)	34 (17.6%)	16 (12.7%)	<.001*
Patients/clients do not know how to use videoconferencing	76 (17.6%)	30 (28.0%)	33 (17.1%)	12 (9.5%)	.001*
Prefer to conduct telephone appointments	71 (16.4%)	42 (39.3%)	23 (11.9%)	6 (4.8%)	<.001*
Prefer to conduct in-person appointments	68 (15.7%)	27 (25.2%)	25 (13.0%)	14 (11.1%)	.005*
Patients/clients do not have access to adequate internet connection	63 (14.6%)	20 (18.7%)	28 (14.5%)	13 (10.3%)	.190
Patients/clients are not interested	52 (12.0%)	15 (14.0%)	26 (13.5%)	11 (8.7%)	.361
Logistics are too complicated (e.g. scheduling an appointment)	44 (10.2%)	29 (27.1%)	11 (5.7%)	3 (2.4%)	<.001*
Have not received training or been instructed to used videoconferencing	39 (9.0%)	14 (13.1%)	18 (9.3%)	7 (5.6%)	.138
Do not own necessary equipment (e.g. webcam)	20 (4.6%)	12 (11.2%)	8 (4.1%)	0 (0.0%)	<.001*
Do not have access to adequate internet connection	18 (4.2%)	9 (8.4%)	5 (2.6%)	4 (3.2%)	.044*
Concerned videoconferencing many not be safe for my patients/clients	10 (2.3%)	5 (4.7%)	3 (1.6%)	2 (1.6%)	.185

* Significant at the *p* < 0.05 level based on *x^2^* analysis between groups.

** Respondents could select all options that apply.

*** Of the respondents to this question *n* = 6 did not declare a profession.

The main aspects of telephone appointments most challenging for respondents were ‘inability to conduct physical exam to the degree required’ (*n* = 256, 95.2%), 95% CI [92.2, 97.4], ‘assess physical health status’ (*n* = 275, 92.3%), 95% CI [88.9, 95.0], and ‘patient's/client's cell phone service is unreliable’ (*n* = 206, 62.0%), 95% CI [56.8, 67.3]. A higher percentage of allied health professionals reported that ‘hearing patients/clients adequately’ was a challenge, as well as ‘patients/clients hearing them adequately’ and ‘establishing rapport with the patient/client’ were challenges (*p* < .05). The key aspects of videoconferencing respondents found most challenging were ‘inability to conduct physical exam to the degree required’ (*n* = 200, 95.3%), 95% CI [90.2, 96.7], ‘patient/client knowing how to use videoconferencing for a virtual appointment’ (*n* = 242, 85.5%), 95% CI [81.3, 89.4], and being able to ‘assess physical health status’ (*n* = 189, 82.9%), 95% CI [77.6, 87.7]. The WhatsApp® platform was reported as the most commonly used videoconferencing platform (*n* = 142, 32.9%), followed by Medcuro® (*n* = 73, 16.9%) and Cisco Webex Meetings® (*n* = 60, 13.9%). A higher percentage of allied health professionals reported using the following platforms for videoconferencing appointments: Google G Suite Hangouts Meet®, Medcuro®, Health Myself (Pomelo)®, Provincial Telehealth System, TELUS Virtual Visits®, WhatsApp®, and Zoom® (standard). A higher percentage of physicians reported using the platform Skype® (standard) for videoconferencing (*p* < .05).

### Relative advantage

**Figure 2:**
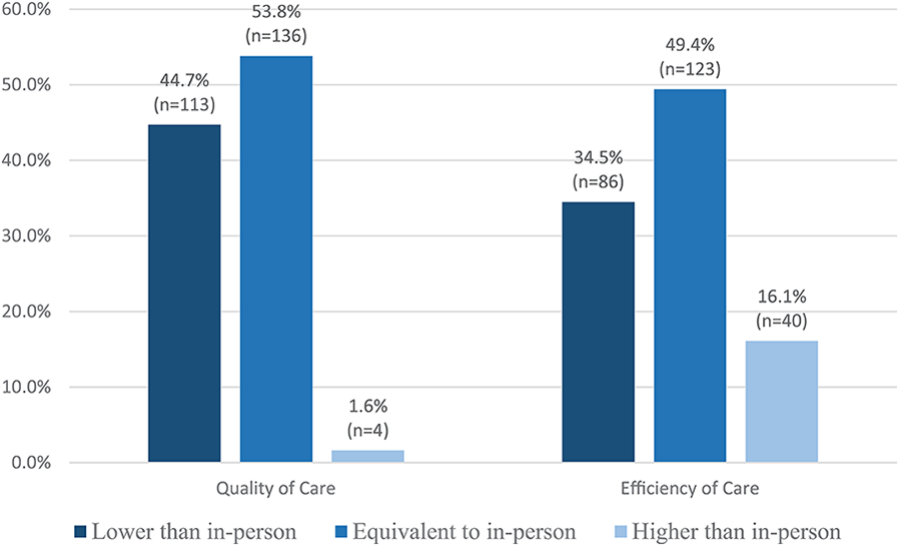
Quality and efficiency of care delivered *via* videoconferencing.

Roger's attribute of ‘relative advantage’ is described as the degree to which an innovation is perceived as better or improves upon existing practices (19). Survey items 19, 20, 22–25 were designed to evaluate experiences and perceptions of the usability of virtual care modalities. Two hundred and forty (*n* = 240, 72.7%) respondents reported being satisfied with telephone appointments, 95% CI [67.8, 77.6], vs. 63.4% (*n* = 164) of respondents satisfied with videoconferencing, 95% CI [57.8, 69.6]. A higher percentage of physicians reported satisfaction with telephone appointments (*p* < .05). Approximately 69% (*n* = 242) reported virtual care has improved their work experience with 57.5% (*n* = 191) reporting quality of care by telephone was lower than in-person, 95% CI [52.3, 62.9], and 42.1% (*n* = 138) reporting efficiency of care by telephone was equivalent to in-person, 95% CI [36.7, 47.3]. A higher percentage of physicians reported that their efficiency of care was higher than in-person with telephone (*p* < .05). One hundred and thirty-six (n = 136, 53.8%) respondents reported the quality of care delivered by videoconferencing was equivalent to in-person, 95% CI [47.8, 60.2]. One hundred and twenty-three (*n* = 123, 49.4%) respondents reported the efficiency of care delivered *via* videoconferencing was equivalent to in-person, 95% CI [42.9, 55.6] (Figure 2). The quality and efficiency of care provided by either telephone or videoconferencing was based on respondent's interpretation.

### Compatibility

All survey respondents were asked to indicate any perceived or actual experienced benefits of virtual care for their practice. Roger's attribute of ‘compatibility’ describes the extent to which an innovation is consistent with the values, past experiences and needs of the potential adopter. Survey item 28 asked respondents to identify the key benefits of virtual care from a checklist of items. The highest rated benefits of virtual care for one's practice included ‘increased patient/client access’ (*n* = 302, 29.8%), ‘ability to work from home’ (*n* = 200, 19.7%), and ‘reduction in no-show appointments’ (*n* = 154, 15.2%) (Table 2). When the ratings for each benefit were compared by professional group using Pearson's *x*^2^ test, the results indicated a significant difference at the *p* < .05 level with a higher percentage of physicians reporting improved relationships with patients/clients, a reduction in no-show appointments, and efficiency as benefits of virtual care.

**Table 2:** Benefits of virtual care by professional group.

Benefits**	All Respondents (*n* = 1013)***	Physicians (*n* = 117)	Nurses (*n* = 552)	Allied Health (*n* = 174)	
	*n* (%)	*n* (%)	*n* (%)	*n* (%)	Sig.
Increased patient/client access	302 (29.8%)	76 (65.0%)	125 (22.6%)	98 (56.3%)	<.001*
Ability to work from home	200 (19.7%)	58 (49.6%)	67 (12.1%)	72 (41.4%)	<.001*
Reduction in no-show appointments	154 (15.2%)	49 (41.9%)	49 (8.9%)	53 (30.5%)	<.001*
Efficiency (e.g. writing notes during the appointment)	129 (12.7%)	47 (40.2%)	45 (8.2%)	35 (20.1%)	<.001*
Improved relationships with patients/clients	92 (9.1%)	38 (32.5%)	35 (6.3%)	17 (9.8%)	<.001*
Increased volume of patient/client appointments/increased revenue	89 (8.8%)	21 (17.9%)	38 (6.9%)	30 (17.2%)	<.001*
Improved specialty provider relationship	53 (5.2%)	12 (10.3%)	27 (4.9%)	13 (7.5%)	.066

* Significant at the *p* < 0.05 level based on *x^2^* analysis between groups.

** Respondents could select all options that apply.

*** Of the respondents to this question *n* = 170 did not declare a profession.

Survey item 26 asked respondents to identify the barriers/challenges they may have experienced with virtual care adoption and/or use. The highest rated barriers or challenges were ‘quality of care/safety’ (*n* = 153, 15.1%), ‘adequate administrative support’ (*n* = 129, 12.7%), and ‘adequate training and education’ (*n* = 94, 9.3%). A higher percentage of physicians reported key challenges as including ‘quality of care/safety’, ‘concerns about increase in demands on time’, ‘lack of integration with current workflow’, ‘concerns about patients/clients overusing services’, ‘work/life balance’, and ‘practice costs to coordinate and conduct’ (*p* < .05). Other barriers or challenges reported by respondents included ‘issues with the technology required for virtual care’, the ‘inability to perform physical and mental health assessments adequately’, and ‘connectivity, poor internet and telephone connections’. The highest rated supports found most useful with integrating virtual care included ‘in-house organizational supports’ (*n* = 149, 14.7%), ‘local colleague support’ (*n* = 122, 12.0%), and ‘technology training on how to use a tool’ (*n* = 114, 11.3%).

Respondents were also asked to rate the importance of a variety of CPD topics on effective use of virtual care. The highest rated CPD topics included ‘comply with regulatory standards/rules for virtual care’ (M = 2.29/3), ‘understand boundaries’ (e.g., personal telephone numbers used to call patients/clients) (M = 2.18/3), and ‘develop and maintain competency and professionalism along continuum while engaging in virtual care’ (M = 2.16/3). A higher percentage of nurses and allied health professionals rated ‘operate virtual care technologies effectively’, and ‘understand ethical challenges of virtual care including access to technology, internet, etc.’ as essential (*p* < .05). Three hundred and ninety-five (*n* = 395, 65.0%) respondents indicated they would be interested in participating in future CPD on virtual care. Respondents were also asked to provide suggestions of topics they would like to see offered in CPD on virtual care. Two hundred and fourteen (*n* = 214) respondents made further suggestions. A thematic analysis was conducted and the topics reported most often by respondents included ‘CPD on how to use the technology’ and the ‘best/easiest platforms for providing virtual care and how to use them effectively’. Another common topic identified by respondents was ‘assessment skills’ and ‘aids for doing assessments virtually’. These assessments included physical, mental, speech language, and cognitive assessments. Ethics, ethical issues (e.g., quality of professional-patient relationship, confidentiality, patient privacy and security of personal health information) and legalities of virtual care were also identified by a number of respondents.

## Discussion

During COVID-19 a variety of virtual care types have been employed with synchronous (telephone and videoconferencing) and asynchronous (e-mail, patient portal messages and e-consults) appointments between patients and providers being the more common modalities (5). Virtual care has also included chatbots, wearable devices and sensors, augmented reality platforms and artificial intelligence (AI) applications such as Apple® health check (5). Contact-tracing applications were also used for COVID-19 prevention and surveillance (14). Based on Roger's notion that attributes of an innovation can be a key determinant in adoption, our survey study sought to explore the role that perceptions of ‘relative advantage’, ‘compatability’ and ‘complexity’ may have played in adopting and using virtual care during the COVID-19 pandemic. Despite the quality of care by telephone being reported as lower than in-person, telephone was the most commonly used method and respondents reported most comfort and satisfaction with telephone appointments. The key benefits reported by respondents in adopting virtual care were increased patient/client access, the ability to work from home, and the reduction of no-show appointments. The quality of care and efficiency provided *via* videoconferencing was rated equivalent to in-person by the majority of respondents, however reasons for not using videoconferencing for virtual care included patients/clients not owning the necessary equipment, or knowing how to use videoconferencing. A number of challenging aspects of conducting both telephone and videoconferencing appointments were reported, including the inability to conduct a physical exam, assess physical health status, patient's/client's cell phone service being unreliable, and patient/client knowing how to use videoconferencing for a virtual appointment. Technology can be a key challenge, particularly with respect to internet connectivity and troubleshooting. Adapting to new technologies for some virtual care modes can also be challenging for workflow integration when compared to in-person appointments, such as connecting with patients virtually for videoconferencing.

Connectivity and familiarity are important for both providers and patients to ensure the effectiveness of virtual care adoption. Providers require training to help them provide virtual care, and patient education can assist in increasing patient understanding of virtual care use and any associated health benefits (3, 5, 13, 17). Additionally, the availability of technical support and adequate resources are useful for providers (5, 8). Physical examination is often an important aspect of the healthcare provider and patient interaction, however virtual care does not easily accommodate traditional in-person examination techniques. Given the practical challenges of conducting physical examinations by telephone or videoconferencing, provider knowledge around how to adapt examination techniques becomes very important for virtual care (8, 10, 17). With virtual care, there is also an increased emphasis for providers to ensure they are meeting an appropriate standard of care and the burden of clinical judgement also becomes heightened, especially during COVID-19 (27). Integration of virtual care can also have an associated increase in administrative tasks for providers and can be difficult to adjust to a new method of conducting appointments with patients (7, 8, 18). The combination of increased responsibilities may also increase risks of burnout for providers (7).

While the survey did not elicit patients’ perceptions, the level of a patient's comfort with technology and lack of technology literacy may also be a barrier for healthcare providers in the adoption and use of particular virtual care modalities. Training and supports are necessary for both healthcare providers and patients. Providers express concerns for the challenges and barriers that patients may experience. Some patients may not have the capability to have a quiet, confidential conversation with a healthcare provider from their home (12, 28). There could be reluctance, or lack of privacy, to use videoconferencing to show certain parts of the body (28). Cultural and generational factors may also influence patients' preferences, comfort and confidence with using virtual care for healthcare services. Patients in some rural areas, or of lower socioeconomic status, may not have the capability to access good internet connectivity, or technology to access virtual care (12, 28). Inadequate internet access can affect the quality of communication between providers and patients, subsequently effecting patient experience (4, 8). Patients unfamiliar with virtual care software could be apprehensive about it, leading to resistance towards virtual care adoption (4, 8, 28). The reality is that many patients experience limited technology literacy and are subsequently unable to use the software adequately (5).

There have been calls for guidelines and recommendations to educate physicians, healthcare providers, and patients on how they can best use virtual care. Due to the increased use of virtual care during COVID-19, a number of guidelines and best practices have been created and distributed publicly. The aim of these resources is to increase knowledge so that virtual care can be confidently integrated into clinical practice. There are fewer resources specific for patients, but those available aim to address lack of familiarity with virtual care. In Canada, work has been done by several groups to develop readily available guidelines for physicians and healthcare providers. The Canadian Medical Association (CMA) and Royal College of Physicians and Surgeons of Canada (RCPSC) have developed the ‘Virtual Care Playbook’ to provide guidance for providers, and connects patients to their ‘Virtual Care Guidelines for Patients’ (29, 30). Canada Health Infoway's ‘Clinician Change Management’ project provides support in the form of virtual care tools and training (31). The Canadian Medical Protective Association (CMPA) also supports providers by providing virtual care informational resources for physicians and healthcare providers through their website (32). British Columbia's Doctors Technology Office has released a ‘Virtual Care Toolkit’ that includes a step-by-step workflow summary for conducting virtual care appointments, including guidelines to enhance privacy and security while employing virtual care (33). Nonetheless, there is a need for further research to explore the specific reasons underlying professionals' perceived ‘quality of care/safety’ issues associated with new modes of virtual care delivery, as well as to more deeply examine the reasons and contexts in which some health professionals prefer to use in-person modes of delivery.

The use of technology necessitates knowledge on how to integrate technology and virtual care in the practice workflow. This includes knowing how to use the technology and the privacy and security of the technology. Providers need to be able to adapt their clinical skills to virtual care and build a rapport through good communication with patients. Virtual care is not appropriate for all visits and providers need to understand when an in-person visit is necessary with respect to the nature of the appointment, as well as contextual factors for individual patients. Finally, providers need to adapt their examination skills to virtual care. Traditional education does not teach providers how to conduct physical exams on a videoconference and lack of training specific to virtual care tools and software, and mobile and wearable health monitoring devices is a challenge for providers. Lack of understanding and training can contribute to provider unwillingness to use virtual care, subsequently challenging virtual care adoption (4, 12, 17, 18). The survey findings also highlight that a majority of respondents were very interested in further CPD on virtual care. Respondents reported that in-house organizational support (e.g., IT supports, quality improvement specialist), local colleague support (e.g., connection with a local colleague who is using the technology), and technology training on how to use a tool (e.g., webinars, recorded videos, one-on-one support) were most useful in supporting integration of virtual care in their practice.

The main limitations to the survey study were the low response rate, the respondent sample being limited to a single province, and the majority of respondents reported working in urban and/or institutional settings, which could limit generalization of the findings to other jurisdictions outside Newfoundland and Labrador. However, the reported adoption rates of virtual care appointment types do reflect fee-for-service billing data and respondents' community of practice results are reflective of general population demographics of the province. This suggests the sample and associated results may be representative of characteristics of the respondent population. As well, the adoption rate of virtual care by physician survey respondents (92.2%, *n* = 107), was also comparable to the findings of the 2021 National Survey of Canadian Physicians (94%). Beyond the COVID-19 pandemic, it is believed virtual care could have a continuing role in enhancing continuity of care in healthcare systems. The survey findings reveal a number of opportunities for supporting healthcare providers in adoption and effective use of virtual care, including supports for adoption and CPD, guidelines and resources to support adaptation to care provision in virtual care environment (e.g., virtual examinations/assessments), as well as patient educational support.

## Data Availability

The raw data supporting the conclusions of this article will be made available by the authors, without undue reservation.

## Footnotes

^1^A restriction policy for people, communities or countries to limit movement and interactions.
