# Supplementary material for: Virtual care and COVID-19: A survey study of adoption, satisfaction and continuing education preferences of healthcare providers in Newfoundland and Labrador, Canada
Source: Front Digit Health. 2023 Jan 25;4:970112. doi: 10.3389/fdgth.2022.970112 (PMC9905429; doi:10.3389/fdgth.2022.970112)
Supplement: Supplementary file 1 [file Table1.docx]

**Supplementary Table 1. Survey respondents’ demographic characteristics**

| **Demographics Characteristics** | **N** | **% of Total Respondents** |
| --- | --- | --- |
| Occupation:  Registered Nurse  Physician  Nurse Practitioner  Nurse Educator  Occupational Therapist  Physiotherapist  Psychologist  Social Worker  Speech Language Pathologist  Other | 509  117  39  15  48  14  23  71  18  23 | 50.2%  11.5%  3.8%  1.5%  4.7%  1.4%  2.3%  7.0%  1.8%  2.3% |
| Specialty:  Family Physician  Specialist | 49  43 | 53.3%  46.7% |
| Population of community practice:  Urban (>10,000)  Small Town (5,000-9,999)  Rural (<4,999) | 555  136  161 | 65.1%  16.0%  18.9% |
| Regional Health Authority:  Eastern  Central  Western  Labrador-Grenfell | 544  119  121  63 | 64.2%  14.0%  14.3%  6.2% |
| Type of Practice:  Solo  Group (2 or more)  Other | 116  322  409 | 13.7%  38.0%  48.3% |
| Please indicate whether your practice is:  Community-based  Institution-based  Other | 334  435  79 | 39.4%  51.3%  9.3% |
| Gender:  Male  Female  Prefer not to disclose  Other | 108  724  12  3 | 12.8%  85.5%  1.4%  0.4% |
| Years in Practice:  0-10 years  11-20 years  More than 20 years | 221  197  431 | 26.1%  23.2%  50.8% |
| Salary Type:  Salaried  Fee-for-service  Self-employed  Contractual  Other | 616  107  35  41  80 | 60.8%  10.6%  3.5%  4.0%  7.9% |
