# Supplementary material for: Virtual care and COVID-19: A survey study of adoption, satisfaction and continuing education preferences of healthcare providers in Newfoundland and Labrador, Canada
Source: Front Digit Health. 2023 Jan 25;4:970112. doi: 10.3389/fdgth.2022.970112 (PMC9905429; doi:10.3389/fdgth.2022.970112)
Supplement: Supplementary file 2 [file Datasheet1.docx]

**Healthcare Provider Survey**

Virtual care has been defined as any interaction between patients/clients and/or members of their circle of care, occurring remotely, using any forms of communication or information technologies with the aim of facilitating or maximizing the quality and effectiveness of patient/client care.1
 
Virtual care can include telephone appointments, videoconferencing appointments, email, secure messaging, and text messaging. Interactions may be provider to patient/client, and provider to provider.


1. Shaw, J., Jamieson, T., Agarwal, P., et al. Virtual care policy recommendations for patient-centred primary care: Finding of a consensus policy dialogue using a norminal group technique. *J Telemed Telecare* 2018; 24(9):608-15.

1. What is the size of the community in which your practice is located?

- Rural ( <4,999)
- Small town (5,000-9,999)
- Urban (>10,000)

2. In which Regional Health Authority is your practice is located?

- Eastern
- Central
- Western
- Labrador-Grenfell

3. What is your practice type?

- Solo physician/practitioner
- Group (2 or more physicians/practitioners)
- Other

4. Please indicate whether your practice is:

- Community-based
- Institution-based
- Other (please specify) ________________________________________________

5. What is your gender?

- Male
- Female
- Prefer not to disclose
- Other: ________________________________________________

6. How many years have you been in practice in Canada?

- 0-5
- 6-10
- 11-15
- 16-20
- 20-25
- 25+

7. Please indicate your occupation. (check all that apply)

- Audiolgist
- Registered Nurse
- Nurse Educator
- Nurse Practitioner
- Occupational Therapist
- Pharmacist
- Physiotherapist
- Physician
- Psychologist
- Social Worker
- Speech Language Pathologist
- Other (please specify) ________________________________________________

*Display This Question:*

*If 7. Please indicate your occupation. (check all that apply) = Physician*

7a. If physician, please indicate your specialty.

- Anatomical Pathology
- Anesthesiology
- Cardiac Surgery
- Child and Adolescent Psychiatry
- Dermatology
- Diagnostic Radiology
- Emergency Medicine
- Endocrinology
- ENT
- Family Medicine
- Gastroenterology
- General Internal Medicine
- General Pathology
- General Surgery
- Geriatric Psychiatry
- Geriatrics
- Gynecology
- Hematology/Oncology
- Hematologic Pathology
- Infectious Disease
- Medical Genetics and Genomics
- Medical Microbiology
- Nephrology
- Neurology
- Neuropathology
- Neurosurgery
- Nuclear Medicine
- Obstetrics and Gynecology
- Ophthalmology
- Orthopedic Surgery
- Otolaryngology-Head and Neck Surgery
- Pediatrics
- Physical Medicine and Rehabilitation Medicine
- Plastic Surgery
- Psychiatry
- Public Health and Prevention Medicine
- Radiology
- Rehabilitation Medicine
- Rheumatology
- Urology
- Vascular Surgery
- Other

8. Please indicate whether you are: (check all that apply)

- Salaried
- Fee for Service
- Self-employed
- Contractual
- Other (please specify) ________________________________________________

9. Do you currently offer any virtual care?

- Yes
- No

*Skip To: Q10 If 9. Do you currently offer any virtual care? = No*

*Skip To: Q12 If 9. Do you currently offer any virtual care? = Yes*

10. If you answered "No" to Question 9, please indicate which of the following reasons why you do not partake in virtual care. (check all that apply)

- My practice did not promote virtual care
- I was not trained on how to provide virtual care
- I tried it and found it frustrating to use
- I did not have office staff to support my virtual care
- I don't believe virtual care is an appropriate way to evaluate patients/clients
- Financial cost
- Scheduling issues
- Lack of technical support
- Lack of financial support
- Concern about being compensated for virtual care
- N/A
- Other (please specify) ________________________________________________

11. If you have not yet, do you plan to implement virtual care in the next 12 months?

- Yes
- No

*Skip To: Q26 If 11. If you have not yet, do you plan to implement virtual care in the next 12 months? = Yes*

*Skip To: Q29 If 11. If you have not yet, do you plan to implement virtual care in the next 12 months? = No*

12. What kinds of virtual care do you offer? (check all that apply)

- Telephone appointments
- Videoconferencing appointments
- Secure messaging
- Regular e-mail
- Text messaging
- Other (please specify) ________________________________________________

13. When caring for patients/clients remotely, do you generally prefer conducting telephone appointments or videoconferencing appointments?

- Telephone
- Videoconferencing

14. If you **DO NOT** conduct videoconferencing appointments, please select the reasons why: (choose all that apply)

- Prefer to conduct telephone appointments
- Prefer to conduct in-person appointments
- Have not received training or been instructed to use videoconferencing
- Logistics are too complicated (e.g. scheduling an appointment)
- Do not own necessary equipment (e.g., webcam)
- Do not have access to adequate internet connection
- Concerned videoconferencing may not be safe for my patients/clients
- Patients/clients are not interested
- Patients/clients do not know how to use videoconferencing
- Patients/clients do not own necessary equipment (e.g., computer, webcam
- Patients/clients do not have access to adequate internet connection
- Other (please specify) ________________________________________________

15. Please rate the following aspects of conducting telephone appointments.

|  | Not a Challenge | Somewhat of a Challenge | Significant Challenge | N/A |
| --- | --- | --- | --- | --- |
| Patient/client knowing how to use a telephone for a virtual appointment |  |  |  |  |
| Patient's/client's cell phone service is unreliable |  |  |  |  |
| Hearing the patient/client adequately |  |  |  |  |
| Patient/client hearing me adequately |  |  |  |  |
| Establishing rapport with the patient/client |  |  |  |  |
| Ensuring patient's/client's safety and confidentiality |  |  |  |  |
| Assessing physical health status |  |  |  |  |
| Inability to conduct a physical exam to the degree required |  |  |  |  |
| Other |  |  |  |  |

16. Please rate the follow aspects of conducting videoconferencing appointments.

|  | Not a Challenge | Somewhat of a Challenge | Significant Challenge | N/A |
| --- | --- | --- | --- | --- |
| Patient/client knowing how to use videoconferencing for a virtual appointment |  |  |  |  |
| Hearing the patient/client adequately |  |  |  |  |
| Patient/client hearing me adequately |  |  |  |  |
| Seeing the patient/client adequately |  |  |  |  |
| Patient/client seeing me adequately |  |  |  |  |
| Establishing rapport with the patient/client |  |  |  |  |
| Ensuring patient's/client's safety and confidentiality |  |  |  |  |
| Assessing physical health status |  |  |  |  |
| Inability to conduct a physical exam to the degree required |  |  |  |  |
| Inappropriate or distracting patient/client behaviour (e.g. patient/client is not properly clothed, in bed, children/pets interrupting the appointment) |  |  |  |  |
| Other |  |  |  |  |

17. Which of the following platforms have you used to conduct videoconferencing appointments? (check all that apply)

- Cisco Webex Meetings / Webex Teams
- Doximity Video
- FaceTime
- Google G Suite Hangouts Meet
- Google Hangouts (standard)
- Go To Meetings
- Jabber
- Maple
- Medcuro
- Microsoft Teams
- Health Myself (Pomelo)
- Provincial Telehealth System
- Skype (standard)
- Skype for Business
- Telus Virtual Visits
- WhatsApp
- Zoom (standard)
- Zoom for Healthcare
- Other (please specify) ________________________________________________

18. What is your comfort level using different modes of virtual care?

|  | Very Uncomfortable | Somewhat Uncomfortable | Neutral | Somewhat Comfortable | Very Comfortable | Not Applicable / Not Using |
| --- | --- | --- | --- | --- | --- | --- |
| Telephone |  |  |  |  |  |  |
| Videoconferencing |  |  |  |  |  |  |
| Secure messaging |  |  |  |  |  |  |
| Email (unsecure) |  |  |  |  |  |  |
| Text messaging |  |  |  |  |  |  |
| eConsult |  |  |  |  |  |  |
| Other (please specify) |  |  |  |  |  |  |

19. If applicable, please rate you satisfaction conducting telephone appointments.

- Very dissatisfied
- Dissatisfied
- Neutral
- Satisfied
- Very satisfied
- N/A

20. If applicable, please rate you satisfaction conducting videoconferencing appointments.

- Very dissatisfied
- Dissatisfied
- Neutral
- Satisfied
- Very satisfied
- N/A

21. Has virtual care improved your work experience?

- Yes
- No

22. How would you rate the "Quality of Care" you deliver via telephone to your patients/clients.

- Lower than in-person
- Equivalent to in-person
- Higher than in-person
- N/A

23. How would you rate the "Efficiency of Care" you deliver via telephone to your patients/clients.

- Lower than in-person
- Equivalent to in-person
- Higher than in-person
- N/A

24. How would you rate the "Quality of Care" you deliver via videoconferencing to your patients/clients.

- Lower than in-person
- Equivalent to in-person
- Higher than in-person
- N/A

25. How would you rate the "Efficiency of Care" you deliver via videoconferencing to your patients/clients.

- Lower than in-person
- Equivalent to in-person
- Higher than in-person
- N/A

26. What are the barriers/challenges that you experience with virtual care? (check all that apply)

- Practice costs to coordinate and conduct
- Quality of care/safety
- Concerns about increase in demands on time
- Work/life balance
- Lack of integration with current workflow
- Adequate training/education
- Adequate administrative support (e.g., comfort with technology, not enough administrative staff to delegate tasks, etc.)
- Concerns about patients/clients overusing services
- Other (please specify) ________________________________________________

27. Are there any supports you accessed and found useful to help you with integrating virtual care in your practice? (check all that apply)

- Local colleague support (Connection with a local colleague who is using the technology)
- In-house organizational supports (e.g., IT support, Quality Improvement Specialists, etc.)
- Change management supports (e.g., workflow integration, defining roles in the team)
- Technical training on how to use tool (webinars, recorded videos, one-on-one support, etc.)
- Written information about how to integrate the tool into workflow
- Evidence about the effectiveness of the tool
- Virtual care standards outlined by my profession's regulatory body
- Written resource on comparison of virtual care platforms (cost, features, proc/cons)
- None
- Other (please specify) ________________________________________________

28. What are the benefits of virtual care for your practice? (check all that apply)

- Increased patient/client access
- Improved relationships with patients/clients
- Improved specialty provider relationship
- Increased volume of patient/client appointments / increased revenue
- Reduction in no-show appointment
- Ability to work from home
- Efficiency (e.g. writing notes during the appointment)
- Other (please specify) ________________________________________________

29. Please rate the level of importance of the following topics to your continuing professional development in effective use of virtual care:

|  | Not Important | Slightly Important | Important | Very Important | Essential |
| --- | --- | --- | --- | --- | --- |
| Recognize limitations of virtual care |  |  |  |  |  |
| Know how virtual care changes approaches to providing patient/client care |  |  |  |  |  |
| Operate virtual care technologies effectively |  |  |  |  |  |
| Know and make use of appropriate clinical exam techniques amenable to virtual care, including substitutes (such as extenders, telemedicine peripherals, home scales, BP monitors, etc.) |  |  |  |  |  |
| Know when to escalate care and how, both within virtual care and beyond |  |  |  |  |  |
| Develop and maintain virtual interview skills and web-side manner |  |  |  |  |  |
| Evaluate and accommodate required accessibility constraints |  |  |  |  |  |
| Plan and adopt virtual care practices according to context of care including geographic location of all involved, distance, collaborating with local care teams, access to interprofessional resources |  |  |  |  |  |
| Understand ethical challenges of virtual care including access to technology, internet etc. |  |  |  |  |  |
| Develop and maintain competency and professionalism along continuum while engaging in virtual care |  |  |  |  |  |
| Comply with regulatory standards/rules for virtual care |  |  |  |  |  |
| Understand boundaries (e.g., personal telephone numbers used to call patients/clients) |  |  |  |  |  |
| Supervise and teach learners: role modelling, creating safe learning environment, assessment and feedback |  |  |  |  |  |

30. Would you participate in any future continuing professional development on virtual care?

- Yes
- No
- Unsure

31. What topics would you like to see offered in continuing professional development on virtual care?

___________________________________________________________________________

_______________________________________________________________________________________________________________________________________________________________________________________________________________________________________________________________________________________________________________________________________________________________________________________

32. Which days of the week is/are most convenient for you to participate in continuing professional development programs? (check all that apply)

- Monday
- Tuesday
- Wednesday
- Thursday
- Friday
- Weekend

33. What time(s) of day is/are the most convenient? (check all that apply)

- Morning
- Afternoon
- Evening

34. What would be your preferred program format(s) for continuing professional development on virtual care? (check all that apply)

- E-Modules (self-paced) / online learning
- Interactive, in-person workshops
- Individual consultations
- Peer consultations
- Interactive webinars
- Other (please specify) ________________________________________________
